# Mixing and matching genes of marine and terrestrial origin in the biosynthesis of the mupirocin antibiotics†

**DOI:** 10.1039/c9sc06192d

**Published:** 2020-05-09

**Authors:** Luoyi Wang, Zhongshu Song, Paul R. Race, James Spencer, Thomas J. Simpson, Matthew P. Crump, Christine L. Willis

**Affiliations:** School of Chemistry, University of Bristol Cantock's Close BS8 1TS Bristol UK chris.willis@bristol.ac.uk matt.crump@bristol.ac.uk; School of Biochemistry, University of Bristol University Walk BS8 1TD Bristol UK; School of Cellular and Molecular Medicine, University of Bristol BS8 1TD Bristol UK

## Abstract

With growing understanding of the underlying pathways of polyketide biosynthesis, along with the continual expansion of the synthetic biology toolkit, it is becoming possible to rationally engineer and fine-tune the polyketide biosynthetic machinery for production of new compounds with improved properties such as stability and/or bioactivity. However, engineering the pathway to the thiomarinol antibiotics has proved challenging. Here we report that genes from a marine *Pseudoalternomonas* sp. producing thiomarinol can be expressed in functional form in the biosynthesis of the clinically important antibiotic mupirocin from the soil bacterium *Pseudomonas fluorescens*. It is revealed that both pathways employ the same unusual mechanism of tetrahydropyran (THP) ring formation and the enzymes are cross compatible. Furthermore, the efficiency of downstream processing of 10,11-epoxy *versus* 10,11-alkenic metabolites are comparable. Optimisation of the fermentation conditions in an engineered strain in which production of pseudomonic acid A (with the 10,11-epoxide) is replaced by substantial titres of the more stable pseudomonic acid C (with a 10,11-alkene) pave the way for its development as a more stable antibiotic with wider applications than mupirocin.

## Abstract 2

Where the sea meets the land: the mupirocin biosynthetic gene cluster (BGC) from the terrestrial bacterium *Pseudomonas fluorescens* was repurposed *via* a plug-and-play approach with heterologous genes from the marine strain that produces thiomarinol.

## Graphical abstract

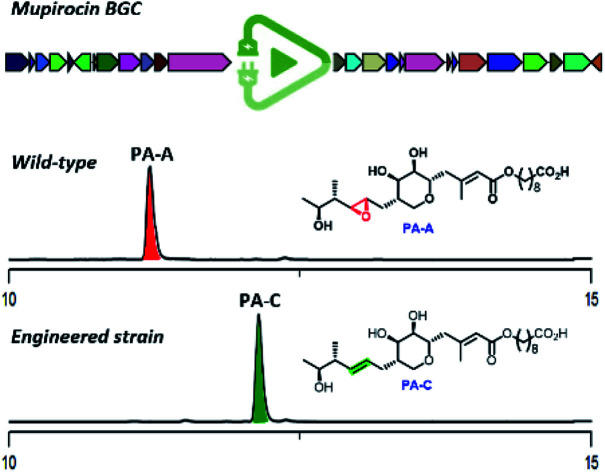

## Introduction

Rapid acceleration in the emergence of antibiotic resistance in pathogenic bacteria is severely eroding our ability to treat bacterial infections.^1^ Central to an effective response to this problem is the development of novel antibiotics with activity against bacteria resistant to existing agents. A majority of antibiotics in clinical use are natural products or their derivatives, possessing chemotypes and properties often lacking in “drug-like” chemical libraries. Thus, rational engineering of natural product biosynthetic pathways for production of new compounds with improved stability and/or bioactivity is an attractive route to overcoming resistance.^2^ However, the success of such approaches requires detailed understanding of the biosynthetic process.^3^

**Fig. 1:**
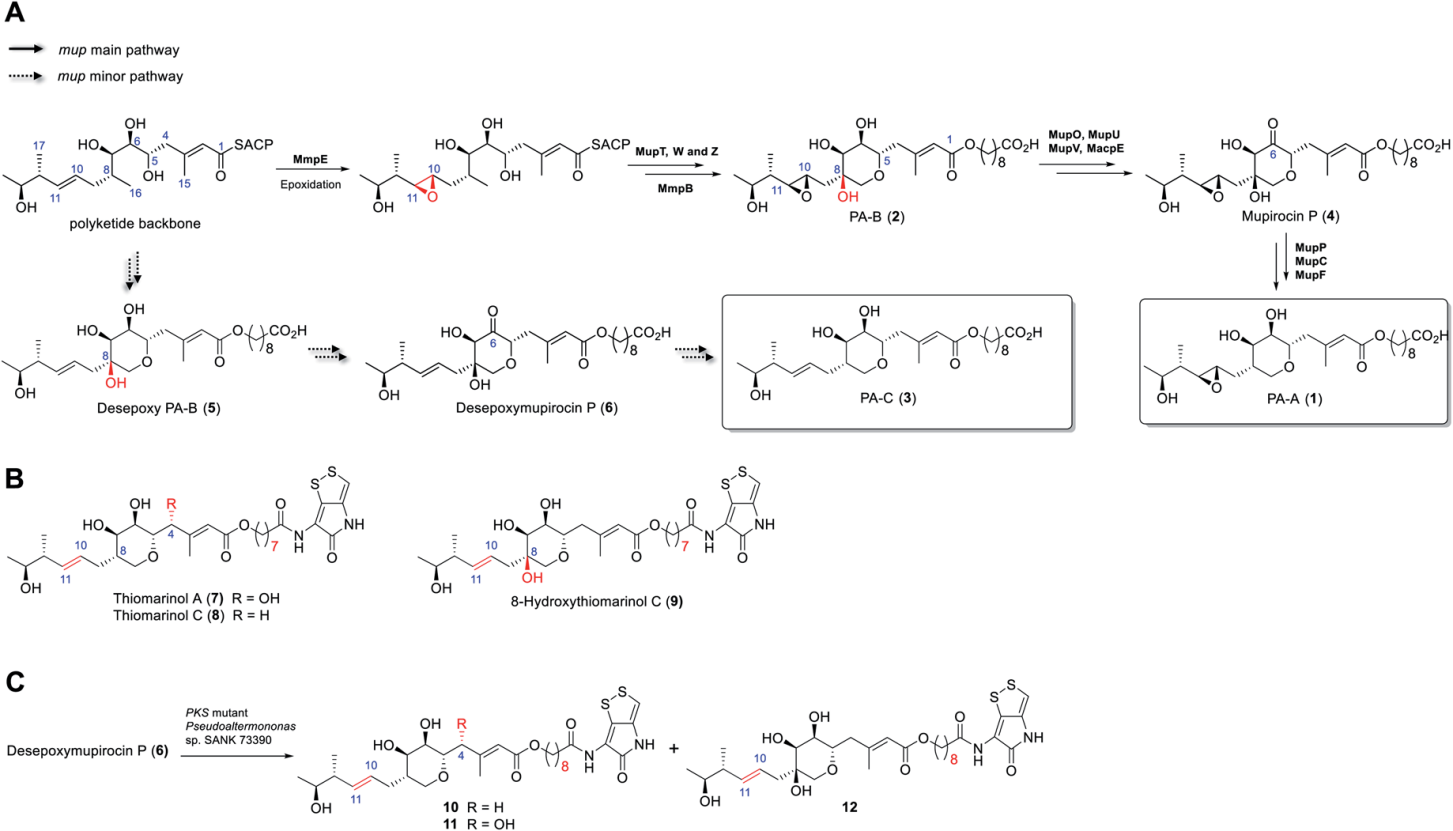
(A) Summary of the later stages of mupirocin biosynthesis showing the parallel pathways. (B) Structures of selected thiomarinols. (C) Transformations of desepoxymupirocin P by the PKS mutant strain of *Pseudoalteromonas* SANK 73390.

The antibiotic mupirocin produced by *Pseudomonas fluorescens* NCIMB 10586 is among the most effective topical treatments for Gram-positive bacterial infections including methicillin resistant *Staphylococcus aureus* (MRSA).^4^ It was identified as one of the first examples of the now extensive family of antibiotics produced by the “*trans*-AT” class of modular polyketides synthases (PKSs).^5^ Mupirocin is a mixture of pseudomonic acids of which the major component (>90%, Fig. 1) is pseudomonic acid A (**1**, PA-A) assembled on a C_17_-polyketide derived moiety (monic acid) esterified by 9-hydroxynonanoic acid (9-HN).^6^ Further minor components of mupirocin include *ca.* 5% PA-B (**2**) containing an additional 8-hydroxyl group and a minor product, <1%, PA-C (**3**), with a 10,11-alkene rather than the 10,11-epoxide found in PA-A and PA-B. Studies on mupirocin biosynthesis including analyses of wild-type and mutant strains of *P. fluorescens* and chemical complementation studies have revealed that PA-B is formed first and contains the tetrahydropyran (THP) ring necessary for activity as shown in Fig. 1A.^7^ The 8-OH is subsequently lost through a series of transformations which begins with oxidation at C-6 to form mupirocin P (**4**), requiring the enzymes MupU (CoA ligase), MupO (P_450_), MacpE (acyl carrier protein, ACP) and MupV (putative didomain oxidoreductase/thioesterase). Dehydration (C7–C8) of **4** catalysed by MupP (dehydratase) followed by two consecutive reductions by the oxidoreductases MupC (C6) and MupF (C7) gives PA-A (**1**).^8^ PA-C (**3**) is the product of a minor parallel pathway that branches from the main pathway (to PA-A).^9^

A practical limitation of the major bioactive component, PA-A, is that the 10,11-epoxide moiety renders the molecule unstable outside a narrow pH range, due to formation of inactive bicyclic products arising from attack of the 7-OH onto the epoxide.^4^ In contrast, the minor metabolite PA-C, containing the 10,11-alkene, is much more stable. It is intriguing that in wild-type *P. fluorescens* 97% of total PAs and related metabolites contain or are derived from a 10,11-epoxide whereas the parallel minor biosynthetic pathway produces <3% of compounds with the 10,11-alkene (*e.g.* PA-C). In contrast, the closely related thiomarinols (*e.g.***7** and **8**) all possess a 10,11-alkene (Fig. 1B).

The thiomarinols are a family of marine natural products isolated from *Pseudoalternomonas* sp. SANK 73390 which also exhibit potent antibiotic activity against MRSA.^10^ They are assembled on a similar C_17_-polyketide moiety to the pseudomonic acids but differ in their esterification with an 8-hydroxyoctanoic acid (rather than 9-HN) side chain further linked as an amide to a pyrrothine subunit of the holomycin class of antibiotics. The major metabolite thiomarinol A (**7**) is also hydroxylated at C-4. Labelling studies are in accord with a PKS/NRPS origin of the thiomarinols.^11^ The mupirocin and thiomarinol gene clusters show significant homology but differences are evident.^12^ For example, homologues of the mupirocin standalone ACP MacpE along with MupU and MupV, necessary for conversion of PA-B to PA-A, are absent from SANK 73390. TmpB however contains additional ketosynthase (KS) and ACP domains as compared to MmpB, which are proposed to replace the apparent missing activities.^12^

Compared with the mupirocin pathway which has been readily modified,^9^ elucidating and engineering of the thiomarinol biosynthetic pathway in the marine organism has proved remarkably challenging. So far, only the *ΔtmlF*, *ΔtmlU*, and the mutant strains of *Pseudoaltermonas* sp. SANK 73390 in which the PKS and NRPS parts of the gene cluster have been deactivated have been successfully generated,^8^ all other gene knock-out studies (*e.g.*, *ΔtmlW*) have failed to produce any isolable metabolites (unpublished results). Characterisation of the family of metabolites, including 8-hydroxythiomarinol C (**9**) (Fig. 1B) isolated from the *ΔtmlF* mutant of *Pseudoaltermonas* sp. SANK 73390, combined with the processing of the THP ring observed on feeding desepoxymupirocin P (**6**) to the *PKS* mutant strain (Fig. 1C),^8^ indicates that it is likely that the late stage biosynthetic machinery generating both thiomarinols and PAs are closely related. However, details of the THP ring formation in thiomarinol biosynthesis remains to be proven experimentally. We now report *in vitro* functional characterisation of key enzymes from the thiomarinol pathway and results from engineering genes from the thiomarinol pathway into the mupirocin system as well as a comparison of the processing efficiency of 10,11-epoxy *versus* 10,11-alkenic metabolites at later stages of the mupirocin biosynthetic pathway. Furthermore, by optimisation of fermentation conditions, a significant increase in titres in cultures of mutants of *P. fluorescens* producing PA-C as the major metabolite has been achieved.

## Results

To investigate the apparent differences in metabolite flux involving intermediates containing the 10,11-epoxide *versus* 10,11-alkenes, we focussed our investigations on two key parts of the mupirocin biosynthetic pathway involving mid and late stage transformations. The latter involves loss of the 8-OH going from PA-B (**2**) to PA-A (**1**) whereas the mid-stage steps occur post PKS-mediated backbone assembly and encompass 10,11-epoxidation, THP ring formation and fatty acid chain extension (Fig. 1A).

First to late stage processing: the conversion of desepoxy-PA-B (**5**) to PA-C (**3**) (*i.e.* loss of the 8-hydroxyl group in metabolites with the 10,11-alkene) was compared with the major pathway involving PA-B with the analogous 10,11-epoxide. PA-B (**2**) was isolated from cultures of the *ΔmupU* mutant of *P. fluorescens* as previously described.^9^ However, a new mutant was required to access the corresponding 10,11-alkene, desepoxy-PA-B (**5**). MmpEOR has been identified as the epoxidase^9^ and thus the double mutant *mmpEΔOR/ΔmupU* was generated and **5** was isolated from fermentations of this strain (see ESI, Fig. S1†) in similar titres to that obtained for PA-B from the *ΔmupU* mutant.

**Fig. 2:**
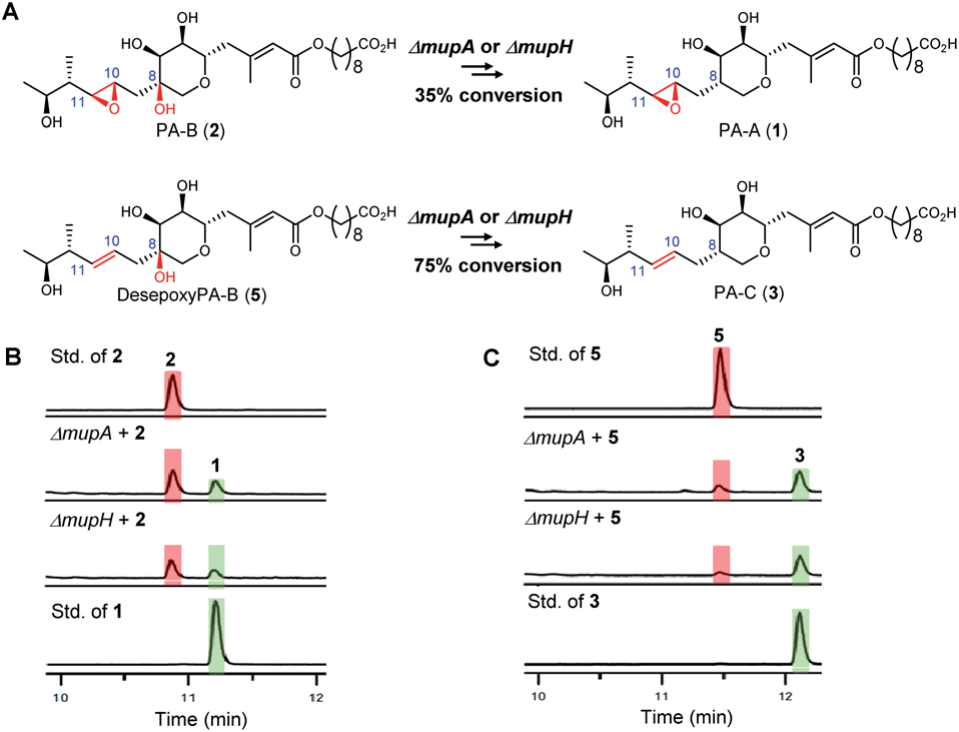
(A) Comparison of the processing of the 8-OH-10,11-epoxy PA (PA-B) with the analogous 10,11-alkene, desepoxy-PA-B. (B) HPLC traces: Conversion of PA-B (**2**) to PA-A (**1**) by the *ΔmupA* and *ΔmupH* mutants. (C) HPLC traces: Conversion of desepoxy-PA-B (**5**) to PA-C (**3**) by the *ΔmupA* and *ΔmupH* mutants.

Two mutant strains, *ΔmupA* and *ΔmupH*, of *P. fluorescens* NCIMB 10586 were selected for the late stage mutasynthesis studies as neither produce any metabolites assembled on 6,7-dihydroxy-tetrahydropyrans.^9^ The same quantity of PA-B (**2**) and desepoxy-PA-B (**5**) was fed to cultures of each of the mutants and after 2 days' growth, products were analysed by LC-MS by comparison with standards for starting materials and products (PA-A **1** and PA-C **3**). Both substrates **2** (35% conversion) and **5** (75% conversion) were efficiently metabolised with overall loss of the 8-OH to PA-A and PA-C respectively (Fig. 2). Thus the steps involved in the removal of the 8-OH are not rate limiting in PA-C production and so do not account for the significantly different titres of PAs and related metabolites containing a 10,11-epoxide (97% of the total) compared with compounds with a 10,11-alkene including PA-C (<3%) in wild-type *P. fluorescens*.

**Fig. 3:**
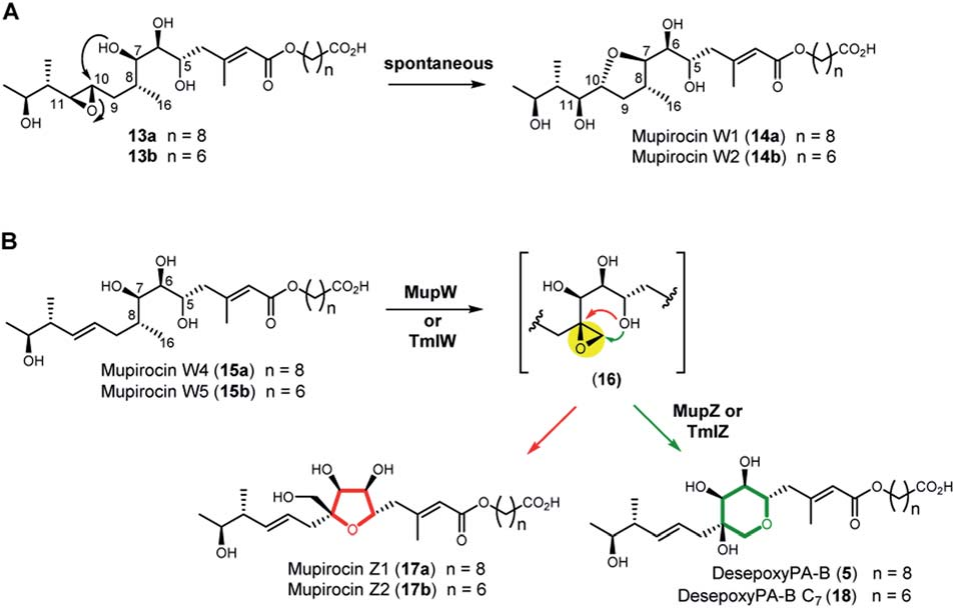
(A) Structures of mupirocin W1 (**14a**) and W2 (**14b**) isolated from the *ΔmupW* mutant of *P. fluorescens*. (B) THP ring formation in mupirocin biosynthesis catalysed by the dual action of the Rieske non-haem oxygenase MupW and the epoxide hydrolase MupZ.^14^ The functions of their equivalents TmlW/TmlZ in thiomarinol biosynthesis were confirmed in the present study.

Next, we turned to the mid-stage of mupirocin assembly and the key step involving tetrahydropyran (THP) ring formation. It has been shown previously that deletion of the oxygenase MupW (or its redox partner MupT) abolished production of the pseudomonic acids and led to accumulation of mupirocin W1 (**14a**) and W2 (**14b**) albeit in low titres.^9,13^ These metabolites lack the THP ring but contain a new tetrahydrofuran ring and are likely shunt products formed from the linear compounds **13a** and **13b** which undergo spontaneous intra-molecular attack by the 7-OH onto the 10,11-epoxide (Fig. 3A).^13^ Thus studies on THP formation on substrates possessing the 10,11-epoxide cannot be undertaken with linear substrates **13a/b**. However, using the corresponding 10,11-alkenes **15a/b** isolated from the *mmpEΔOR/ΔmupW* strain we have recently shown that THP formation occurs *via* an oxidative enzyme-catalysed cascade by the dual action of the Rieske non-haem oxygenase MupW and the epoxide hydrolase MupZ.^14^ Selective oxidation of the C8–C16 single bond in the acyclic precursors mupirocin W4 and W5 (**15a/b**) is proposed to give the corresponding 8,16-epoxide **16** (Fig. 3). In the absence of MupZ, the five-membered tetrahydrofuran ring products mupirocin Z1 and Z2 (**17a/b**) were isolated *via* spontaneous cyclisation (Fig. 3B).^14^

TmlW and TmlZ from the thiomarinol gene cluster, show 55.8% and 56.3% identity, respectively, to MupW and MupZ. To investigate if the same mechanism of tetrahydropyran formation operates in both thiomarinol and mupirocin biosynthesis, TmlW was heterologously overexpressed in *E. coli* which is able to provide the additional redox partners and cofactors necessary to constitute an active cascade.^14^ Linear substrate mupirocin W5 (**15b**) was added to a whole-cell system using resting TmlW overexpressing *E. coli* cells supplemented with glucose to enable NAD(P)H regeneration (ESI†). LC-MS indicated full conversion to a new product had occurred which on isolation and analysis by NMR spectroscopy was confirmed to be tetrahydrofuran mupirocin Z2 (**17b**), identical to that previously obtained from the whole live cells of the MupW overexpressing *E. coli* strain (Fig. S2†). Furthermore, incubation of mupirocin W4 (**15a**) with *E. coli* cells overexpressing both MupW and TmlZ afforded the THP ring product desepoxy-PA-B (**5**) (Fig. S2†). These results not only confirm that thiomarinol biosynthesis employs the same THP ring forming mechanism as that of mupirocin, but also demonstrate that these enzymes are cross compatible.

**Fig. 4:**
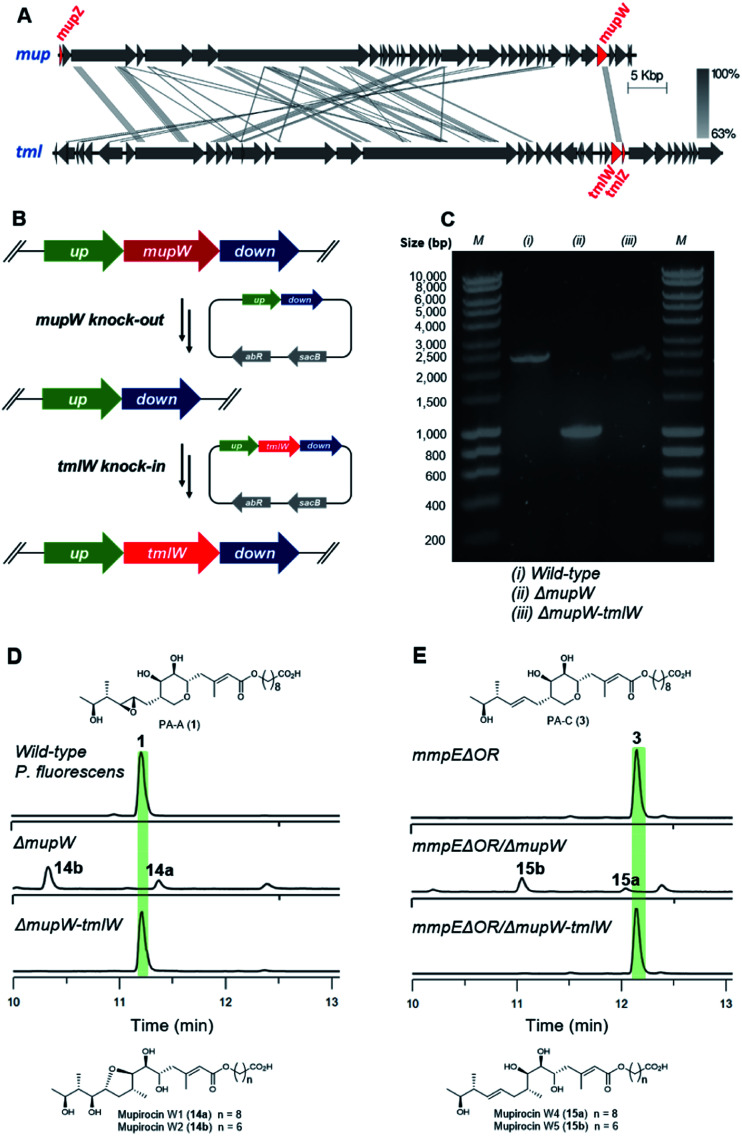
(A) Comparison of the mupirocin (*mup*) and thiomarinol (*tml*) biosynthetic gene cluster, key genes involved in THP ring formation are highlighted in red, *mupW* is replaced by *tmlW* to form the chimeric pathway in this study. (B) Gene knock-out and knock-in by the two-step allelic exchange method. (C) PCR identification of the knock-out and knock-in mutants. (D) HPLC traces of the wild-type, *ΔmupW* and *ΔmupW-tmlW* mutants of *P. fluorescens*. (E) HPLC traces of the *mmpEΔOR*, *mmpEΔOR/ΔmupW* and *mmpEΔOR/ΔmupW-tmlW* mutants of *P. fluorescens*.

Unlike the major metabolites from wild-type *P. fluorescens* which contain the 10,11-epoxide, all thiomarinols isolated from *Pseudoalteromonas* sp. SANK 73390 have a 10,11-alkene. Having demonstrated MupW/TmlW and MupZ/TmlZ are functionally interchangeable we therefore investigated if replacing *mupW* with *tmlW* in the mupirocin gene cluster (Fig. 4A) would lead to more efficient processing of 10,11-alkenes *versus* 10,11-epoxides and hence alteration of the PA-C (**3**) to PA-A (**1**) ratio.

The entire *mupW* gene was firstly knocked out from the wild-type strain of *P. fluorescens* using the two-step allelic exchange method (Fig. 4B).^14,15^ Desired mutants were screened by PCR and confirmed by Sanger sequencing (Fig. 4C). The resulting *ΔmupW* mutant produced only the THF-containing shunt products mupirocin W1 and W2 (**14a/b**) (Fig. 4D). *TmlW* was then introduced into the *ΔmupW* strain at the same locus from which *mupW* was removed, using the same genetic manipulation approach. This new hybrid mutant, *P. fluorescens ΔmupW-tmlW*, was cultured in parallel with the original wild-type *P. fluorescens* under the same fermentation conditions. This restored PA-A production and the ratio of PA-C (trace) to PA-A (major) in cultures of the mutant strain was equivalent to that obtained for wild-type *P. fluorescens* (Fig. 4D).

Next, the same knockout–knockin approach was applied to the *mmpEΔOR* mutant of *P. fluorescens* which only produces PA-C. The *mmpEΔOR/ΔmupW* mutant abolished PA-C production and gave only the acyclic metabolites mupirocin W4 and W5 (**15a/b**) (Fig. 4E). The new hybrid mutant *mmpEΔOR/ΔmupW-tmlW* restored PA-C production, and titres were similar as compared to *mmpEΔOR* (Fig. 4E).

**Fig. 5:**
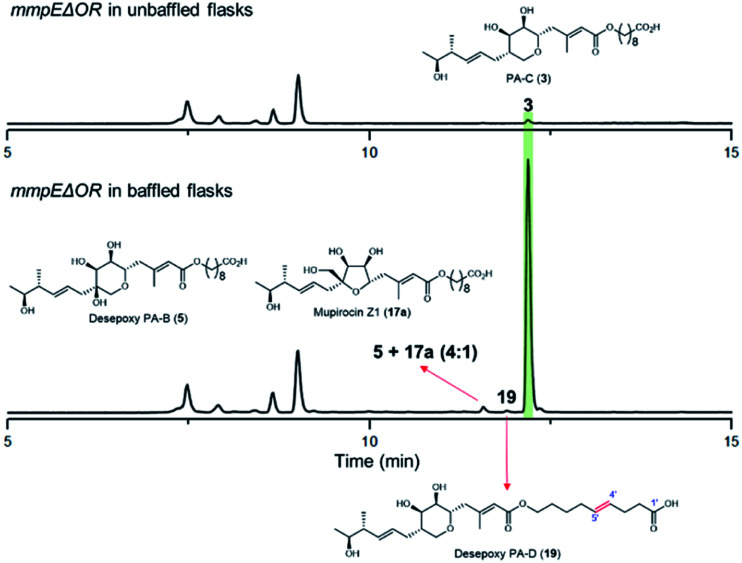
HPLC trace of the *mmpEΔOR* mutant cultured in baffled flasks compared to in unbaffled flasks.

During our investigations we found that the titres of mupirocin metabolites from both wild-type *P. fluorescens* and mutants were variable and hence we undertook a systematic investigation to improve both the reproducibility and importantly the levels of metabolite production. Fermentation media, incubation time and temperature, shaking speed *etc.* were optimised for PA-C production. The key observation was that when the *mmpEΔOR* mutant was grown for 2 days at 22 °C in LB-broth supplemented with 4% glucose, the titres of PA-C (**3**) reached a reproducible level of *ca.* 40 mg L^−1^ in baffled flasks compared to *ca.* 2 mg L^−1^ in unbaffled flasks (Fig. 5 and S3†). Minor metabolites isolated under these conditions include a 4 : 1 mixture of desepoxy-PA-B (**5**) with mupirocin Z1 (**17a**) (Fig. 5 and S4†), and the newly identified desepoxy-PA-D (**19**) (Fig. 5, Table S1 and Fig. S5–S10†). The new hybrid mutant *mmpEΔOR/ΔmupW-tmlW* showed a similar result, again indicating their equivalent effectiveness in initiating THP ring formation. Interestingly, increased aeration during fermentation of cultures of mutants acting early in the pathway led to no detectible differences in titres of accumulated acyclic intermediates such as **15a** and **15b**.

With significant quantities of PA-C now available, antibiotic activity screening of PA-C, along with PA-A and PA-B, against a range of Gram-positive bacteria (Table 1) was undertaken. The more chemically stable PA-C (**3**) exhibited comparable antibiotic activity to PA-A, with, notably, similar activity against the four *Staphylococcus aureus* strains tested, including the Mu50 strain that both exhibits a methicillin-resistant (MRSA) phenotype and reduced susceptibility to glycopeptides including vancomycin. These data confirm the effectiveness of monic acid-based agents against Gram-positive pathogens, including strains challenging to treat with first-line antibiotics, and that antibacterial activity is not adversely affected by substitution of the 10,11-epoxide of mupirocin for the 10,11-alkene. Notably, PA-B displayed substantially lower activity in all susceptibility assays. As PA-B and desepoxy-PA-B are normally intermediates towards the mature antibiotics PA-A and PA-C, respectively, this may be of physiological significance in terms of self-resistance.

**Table 1:** Minimum Inhibitory Concentration (MIC, μg mL^−1^) of PA-A (**1**), PA-B (**2**) and PA-C (**3**) against Gram-positive bacteria. Values were obtained from microplate broth dilution

Strains	PA-A (**1**)	PA-B (**2**)	PA-C (**3**)
*Bacillus subtilis*	0.06	1	<0.03
*S. aureus* ATCC 29213	0.125	4	0.25
*S. aureus* Newman	0.125	2	0.125
*S. aureus* NCTC 6571	0.125	2	0.125
*S. aureus* Mu50	0.125	2	0.125
*S. epidermidis* NCTC 11047	0.125	4	0.5

## Conclusions and discussion

In conclusion we have demonstrated that the late-stage processing in mupirocin biosynthesis of the 10,11-alkene is as efficient as the 10,11-epoxide and that transformations leading to the removal of the 8-hydroxyl groups do not account for the very different levels of PA-A and PA-C production in WT *P. fluorescens*. Epoxidation catalysed by MmpE is presumably very efficient. The mupirocin pathway was engineered with heterologous genes from the thiomarinol pathway thereby demonstrating that genes from the marine organism *Pseudoalternomonas* sp. SANK 73390 can be expressed in functional form in the soil bacterium *P. fluorescens*. Furthermore, it is revealed that both pathways employ the same unusual mechanism of tetrahydropyran (THP) ring formation and that the enzymes responsible for this activity are cross compatible.

Whilst the mupirocin and thiomarinol gene clusters show high homology, there are significant differences.^12^ Genetic engineering of the thiomarinol pathway in the marine organism has, however, proved remarkably difficult (only four mutants have been reported to date^8^), leading to significant challenges in elucidating the biosynthesis of these marine natural products. We envisage that heterologous expression of components from the thiomarinol pathway in the mupirocin producer will provide an alternative approach elucidating their biosynthesis.

Access to engineered strains of *P. fluorescens* in which the major product is the more stable PA-C, rather than PA-A in wild type, has potential for industrial scale production of PA-C and subsequent further development as a more stable antibiotic with wider applications than mupirocin.

The plug-and-play approach for the assembly of heterologous genes in the mupirocin biosynthetic gene cluster will be useful for further engineering of polyketide biosynthesis. By building on existing polyketide engineering and characterisation strategies, harnessing current synthetic biology technologies, and utilising advances in metabolic/host engineering, we anticipate future successes in engineering PKSs capable of producing designer polyketides for applications in medicine, fuels, and industrial products.^16^

## Conflicts of interest

There are no conflicts to declare.

## Supplementary Material

SC-011-C9SC06192D-s001
