# Supplementary material for: Mixing and matching genes of marine and terrestrial origin in the biosynthesis of the mupirocin antibiotics
Source: Chem Sci. 2020 May 9;11(20):5221–6. doi: 10.1039/c9sc06192d (PMC8159325; doi:10.1039/c9sc06192d)
Supplement: SC-011-C9SC06192D-s001 [file SC-011-C9SC06192D-s001.pdf]

Electronic Supplementary Material (ESI) for Chemical Science.
This journal is © The Royal Society of Chemistry 2020

Supporting Information

Mixing and Matching Genes of Marine and Terrestrial Origin in the Biosynthesis of the Mupirocin Antibiotics

Luoyi Wang,[a] Zhongshu Song,[a] Paul R. Race,[b] James Spencer,[c] Thomas J. Simpson,[a] Matthew P. Crump, *[a] Christine L. Willis*[a]

[a] School of Chemistry, Cantock's Close, University of Bristol, BS8 1TS, Bristol, UK

[b] School of Biochemistry, University Walk, University of Bristol, BS8 1TD, Bristol, UK

[c] School of Cellular and Molecular Medicine, University of Bristol, BS8 1TD, Bristol, UK

*E-mail: chris.willis@bristol.ac.uk; matt.crump@bristol.ac.uk

1. General experimental procedures

LC-MS data were obtained on a Waters LCMS system comprising Waters 2767 autosampler, Waters 515 HPLC pump, Waters 2998 Diode Array detector, Waters 2424 ELS detector and Waters Quatro Micro mass spectrometer. HPLC grade H_2O and MeCN were added with 0.05% formic acid as solvent system. Analytical LC-MS data were obtained using a Phenomenex Kinetex column (C_{18}, 250 x 4.60 mm, 5 µm) at a flow rate of 1 mL/min, with a gradient of 5% MeCN to 95% MeCN in 20 mins. Preparative HPLC purification were carried out using a Phenomenex Kinetex column (C_{18}, 250 x 21.20 mm, 5 µm) at a flow rate of 16 mL/min. HRESIMS data were obtained on a Bruker Daltonics micrOTOF II instrument. NMR data were collected on Bruker Cryo500 or Bruker Cryo700 Micro-Coil NMR spectrometers. Antibacterial susceptibility testing was carried out by the microbroth dilution method in cation-adjusted Mueller-Hinton broth according to CLSI guidelines[1].

2. Construction of mutant strains of *Pseudomonas fluorescens* NCIMB 10586

Synthetic DNAs for *mupW* knockout and *tmlW* knockin were purchased from Thermo Fisher Scientific, containing approximately 500 bp overlapping fragments upstream and downstream of the target gene and 15 bp appropriate homologous sequences to suicide vector pEX18Tc at both ends.

mupW-KO:

acgaattcgagctcgTTTCACAGTCGCGTAGCCAGGCAGAGCGCTTTCACGCGTCGTTTTTTGCGCGCTCCGGCAGTGAGAATA
CCGAGAGTGCGACACAGAGCCGCCAATCCAGCGAGATCCTCGACGCGACGGATCCGAACCTTATCCACCTGACGAGCCT
GCCCTTTGAATCCGCGGAGTCGCTGTATTGCTATAGCCGGGCTATTACCGCGCTGCAAGTCGGCGAGTACCCGCTGCAGG
ATGAGCAACTCGGGGTGCCGTGCCATGTCATCGCCGGCACCTGTGATGAAGCTGTGAGCCCCCATGAGTCAAAGCGCTTT
ATCGAACTGCATGAGGGTGCTGACTATTCGGAAATCGCTGGCATGGATCATTTTGGTTTGTATAACCACCTGGGGTTGTTG
GGGGTGGTGACGGCGTATATCAAGAACCTCAGCCCGGTGATGCCGGGTTCCCAGGCGGATGCATAAGTCCAGCCTGCTC
ATCCACACGGCATCAAAGGAGGGAAGACCATTCGTCACGGGACTTAATGCGCGCCATTATGACTTTTGTTTAATTTGACAA
TCTATGTTTTTTCGTTAACTTACTTCTTGAAGTGGATTGGATCCAACCTGCCTGGCAAGGTGAGCGTTGCTCTTCGAATAGT
GGCCATTCAATGTGGGGCGGCCACTAATAGTCCCCATATAACAAGGAGGTTAAGATGCTTGAAGACATTCTGATGTGTGA
GACGCTGGAGGCCTGGAGTAAAGTGCTGTTTGCCTTGGCGAAAGAGTATGGATTTTCTTCGGTGCTGTTTGGGGTTAAGC
CCACGATCAGTACGCCTTTCAGCTCTTCGACCATCATCAGTAATTATTCCAGGTCATGGCGCAGTATCTATGACGCGAATG
CCTATTACGAAGTCGACCCTGTTGTGTTTCATTGCTTGAACAGTTCATTGCCGTTGGTCTGGACAAAGGAAAATTTTATTAA
CAAGCCCGAACAGCAATTGTATGAGGCTGCGAGagcttggcactggcc

tmlW-KI:

acgaattcgagctcgTTTCACAGTCGCGTAGCCAGGCAGAGCGCTTTCACGCGTCGTTTTTTGCGCGCTCCGGCAGTGAGAATA
CCGAGAGTGCGACACAGAGCCGCCAATCCAGCGAGATCCTCGACGCGACGGATCCGAACCTTATCCACCTGACGAGCCT
GCCCTTTGAATCCGCGGAGTCGCTGTATTGCTATAGCCGGGCTATTACCGCGCTGCAAGTCGGCGAGTACCCGCTGCAGG
ATGAGCAACTCGGGGTGCCGTGCCATGTCATCGCCGGCACCTGTGATGAAGCTGTGAGCCCCCATGAGTCAAAGCGCTTT
ATCGAACTGCATGAGGGTGCTGACTATTCGGAAATCGCTGGCATGGATCATTTTGGTTTGTATAACCACCTGGGGTTGTTG
GGGGTGGTGACGGCGTATATCAAGAACCTCAGCCCGGTGATGCCGGGTTCCCAGGCGGATGCATAAGTCCAGCCTGCTC
ATCCACACGGCATCAAAGGAGGGAAGATGAATAAATTGGCACCGAAAAACGGCACATACACAAGCTATCAGGATGTGAT
TGCAACTGATAAGATAACTCCGCCCGAGGACATGCATATACATTCTTCGGTACAATACAACTTTCACTCTATCAGTAAGGC
ACGTTACACCGAGCCTGAATATCATGATTTAGAGATGAAAAAAGTTTGGTCACGGGTTTGGCAATTGGCCTGCCGTGTTG
AAGATATTCCTGAGAAGGGAGACACATTTGTTTACGAGATTGGTCATTTATCATTGATCATCGTTCGGGTCTCAGATGATG
AACCTCAGAATCTGGATGAAACAAAAGTGATCAAGGCGTACTATAACTCATGCCGCCACCGAGGACGAAAACTGATTGA
GCACAATACTAGTGTGCAATGTATTCGGTGTCCATATCATGGTTTTACATGGTCATTGGATGGTGAACTGAGCGAAATCCC
CTATGAATGGGATTTTTCAAAAATAGAAAAGAAGTCAATGCCGCTGTTGGAAGTACGTGTCGATGTGTGGGATGGGTTTG
TATTCATTAATATGGATCCTGACGCAGAGACTTTAAAATCCTACTTAGAAATTTTGCCCGATCAGCTTGCTAACAACTTCTA
TGGTGAGAAAGTGGTGGTAAAACATAGTGAACAAGTTTTGCCAGCAAATTGGAAAACGGTCATGGAATCTTTTATGGAG
GGATTGCATGCACAAGAAACTCACGGACACACATGGCCGCACCTAAGTGATATATTGCAATATGATACATTTCCAGATGTT
CGGCACATATCAAGAAGTTTTCATGCAGTGGGCTTGCAAGTGAGCGAAGGGCGTGAGAAGTTGACAGAGCAAGAGATCA
TGGATCACTTTCACCATAATGTACATTTAGGCAACCCAAATACGGCACCTCCTCAGCTTGGCTCTGGAGTAACCGCAAGGG
AGTACATGGCTGACATTATGCGTGCCCAACATACCTTGAAAACGGGCAAGGATCATATGCATATCTCAGATGCCGAAGCA
TTAGATGTATTGCAGTACACGCTATTTCCAAACATCATTTTGTTTCGTGGTATCACTCTGCCACTTATTTTACGATTTAGACC
CCATAAGAATGATCACAGCCAGTGTATTTTCGACATCTTCTATTTAGAAGATAAACCGAAAAATCAAACTGCTTATTCCCCT
GCTGAGACGATTCATATGGCTAAAGGCGATACATATGAAGAAGCGGGTGTACTGGAGGATTGGTTAGGTCATGTCTACG
ATCAAGATATGGTTAATATTGCAAATATGCATGGTGGCTTGCTTTGTGGGCCCGATCAAGAAGTGTACCTCAGTGACTATC
ATGAGTCTCGGATCAAACACTTTCACCATGCATTGGACCTTTATTTAAACAAAGAAAGCTAGACCATTCGTCACGGGACTT
AATGCGCGCCATTATGACTTTTGTTTAATTTGACAATCTATGTTTTTTCGTTAACTTACTTCTTGAAGTGGATTGGATCCAAC
CTGCCTGGCAAGGTGAGCGTTGCTCTTCGAATAGTGGCCATTCAATGTGGGGCGGCCACTAATAGTCCCCATATAACAAG

GAGGTTAAGATGCTTGAAGACATTCTGATGTGTGAGACGCTGGAGGCCTGGAGTAAAGTGCTGTTTGCCTTGGCGAAAG
AGTATGGATTTTCTTCGGTGCTGTTTGGGGTTAAGCCCACGATCAGTACGCCTTTCAGCTCTTCGACCATCATCAGTAATTA
TTCCAGGTCATGGCGCAGTATCTATGACGCGAATGCCTATTACGAAGTCGACCCTGTTGTGTTTCATTGCTTGAACAGTTC
ATTGCCGTTGGTCTGGACAAAGGAAAATTTTATTAACAAGCCCGAACAGCAATTGTATGAGGCTGCGAGagcttggcactggc
c

DNA fragment containing ca. 500 bp upstream and downstream of *mupU* was amplified from *ΔmupU* mutant of *P. fluorescens* NCIMB 10586 for the purpose of *mupU* knockout in the *mmpEΔOR* mutant. These DNA fragments were introduced into KpnI/HindIII-digested pEX18Tc by In-Fusion HD Cloning Kit, yielding allelic exchange suicide vectors pEX18Tc-mupUKO, pEX18Tc-mupWKO and pEX18Tc-tmlWKI, respectively, which were then transformed into *E. coli* S17.1 (λ *pir*$^+$). 1.5 ml of the overnight *E. coli* donor strain containing the suicide plasmids and 0.5 ml of the wild-type or *mmpEΔOR* mutant of *P. fluorescens* NCIMB 10586 recipient strain were centrifuged at 10,000*g* for 5 min at room temperature. Each cell pellet was collected and resuspended in 50 µl of LB and then combined. This cell mixture was transferred onto the middle of a prewarmed LB agar plate. The 'puddle' was allowed to dry on the agar surface and incubated overnight at 30 °C. The mating puddle was then scraped off and resuspended in 1.0 ml of sterile saline solution. 50-, 200-, and 500-µl aliquots were spread on LB agar plates supplemented with tetracycline (15 µg/ml) and carbenicillin (50 µg/ml) and incubated for 72 hours at 30 °C. Single colonies were picked from above LB plates and streaked onto no-salt LB + 15% (wt/vol) sucrose agar, followed by incubation for 48 hours at 30 °C. Colonies were screened for the targeted knockout or knockin mutants by PCR using the upstream forward and the downstream reverse primer pair.

mupU-Up-For: 5'-CGACCAGATCGTCCAGTGGT-3'

mupU-Down-Rev: 5'-GTGCTCTGGTGCAGGCATTC-3'

mupW-Up-For: 5'-TTTCACAGTCGCGTAGCCAG-3'

mupW-Down-Rev: 5'-CAATTGTATGAGGCTGCGAG-3'

3. General fermentation procedure for wild-type and mutant strains of *P. fluorescens* NCIMB 10586

Wild-type and mutant strains of *P. fluorescens* NCIMB 10586 were inoculated on L-agar plates (1% Bacto tryptone, 0.5% yeast extract, 0.5% sodium chloride, 0.1% glucose, 1.5% agar) and incubated overnight at 30 °C. Seed medium was inoculated in a 50 mL Falcon tube with 10 mL of L-broth (1% Bacto tryptone, 0.5% yeast extract, 0.5% sodium chloride, 0.1% glucose) by picking a single colony from the L-agar plate and incubated overnight at 200 rpm at 30 °C. Production fermentation was inoculated with 5% of seed culture in 50 mL of L-broth with 4% w/v glucose in a 250 mL 1-baffled flask. The culture was incubated at 22 °C at 200 rpm for 2 days and then centrifuged at 8000 rpm for 15 mins. The supernatant was extracted with EtOAc 3 times and the combined EtOAc extracts were evaporated *in vacuo* to give a crude extract, which was subjected to LC-MS analysis or further purification.

4. Isolation of desepoxy PA-B (5) from *mmpEΔOR/ΔmupU* of *P. fluorescens* NCIMB 10586

A 150 mL scale fermentation of *mmpEΔOR/ΔmupU* of *P. fluorescens* NCIMB 10586 was carried out as per general procedure. The crude extract (Figure S1) was purified by HPLC eluted with a gradient of 30 to 70% of MeCN in water over 20 minutes to yield desepoxy PA-B (**5**, 7.0 mg).

5. Feeding of PA-B (2) and desepoxy PA-B (5) to *ΔmupA* and *ΔmupH* of *P. fluorescens* NCIMB 10586

A 50 mL scale fermentation of *ΔmupA* and *ΔmupH P. fluorescens* NCIMB 1058 was carried out as per general procedure. 0.25 mg of PA-B (isolated from the *ΔmupU* strain) or desepoxy PA-B (isolated from the *mmpEΔOR/ΔmupU* strain) dissolved in 50 µL of MeOH was fed to *ΔmupA* or *ΔmupH* of *P. fluorescens* NCIMB 10586 immediately after inoculation. After 2 days of fermentation, the culture was extracted with EtOAc to give a crude extract, which was subjected to LC-MS analysis.

6. Whole-cell biotransformation with TmlW and TmlZ

The *tmlW* and *tmlZ* genes were amplified from *Pseudoalternomonas* sp. SANK 73390 genomic DNA by PCR and then introduced into pET28a vector (pre-linearized with EcoRI and SacI), and pOPINF (pre-linearized with KpnI and HindIII), respectively, using the In-Fusion HD Cloning Kit (Clonetech). The resultant plasmids were verified by sequencing and transformed into *E. coli* BL21 (DE3). Expression of TmlW and co-expression of MupW and TmlZ were carried out with auto induction medium LB broth base including trace elements (FORMEDIUM™). In *in vivo* biotransformations, 50 mL of overnight culture of *E. coli* BL21 (DE3) cells overexpressing TmlW or both MupW and TmlZ were centrifuged at 6000 rpm for 5 min, cell pellets were resuspended in 2 mL of 100 mM potassium phosphate buffer pH 7.2 supplemented with 20 mM glucose, substrates dissolved in MeOH were then added and the reactions were incubated at 30 °C, 180 rpm for overnight. Reactions were quenched by adding equal volume of acetonitrile, vortexed and centrifuged. The acetonitrile layer was injected for LC-MS analysis and products were purified by preparative HPLC for NMR analysis (Figure S2).

7. Fermentation of *mmpEΔOR* of *P. fluorescens* NCIMB 10586 and quantification of PA-C (3)

Fermentation of the *mmpEΔOR* mutant of *P. fluorescens* NCIMB 10586 was carried out as per general procedure. Quantification of PA-C (**3**) was carried out using the calibration curve method. Standard solutions with different concentrations of PA-C (0.001-0.15 mg/mL in MeOH) were prepared from a stock solution of PA-C (1.0 mg/mL in MeOH) by serial dilutions. The same injection volume (50 µL) of these samples, along with crude extract samples from fermentation, were analysed by LCMS and the peak areas for PA-C from the diode array traces were recorded. Microsoft Excel is used to plot the peak area against concentration for a standard curve and an equation of y = 3E+07x + 12031 (R^2 = 0.9998) was generated (Figure S3), which was then used to calculate the PA-C concentrations in unknown samples.

For isolation of minor metabolites obtained under this condition, a 400 mL scale fermentation was carried out and the crude extract was purified by HPLC eluted with a gradient of 40 to 50% of MeCN in water over 20 minutes to yield 1.2 mg of a 4:1 mixture of desepoxy PA-B (**5**) with mupirocin Z1 (**17a**) (Figure S4), and 0.5 mg of desepoxy PA-D (**19**) (Table S1, Figure S5-S10).

References

1. CLSI. Methods for Dilution Antimicrobial Susceptibility Tests for Bacteria That Grow Aerobically. 11th ed. CLSI standard M07. Wayne, PA: Clinical and Laboratory Standards Institute, 2018.

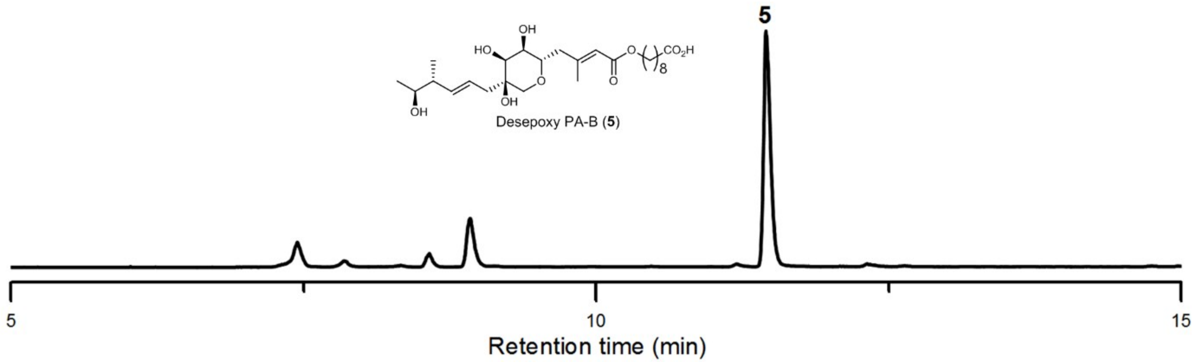

Figure S1. HPLC trace of the crude extract of the *mmpEΔOR/ΔmupU* double mutant of *P. fluorescens* NCIMB 10586

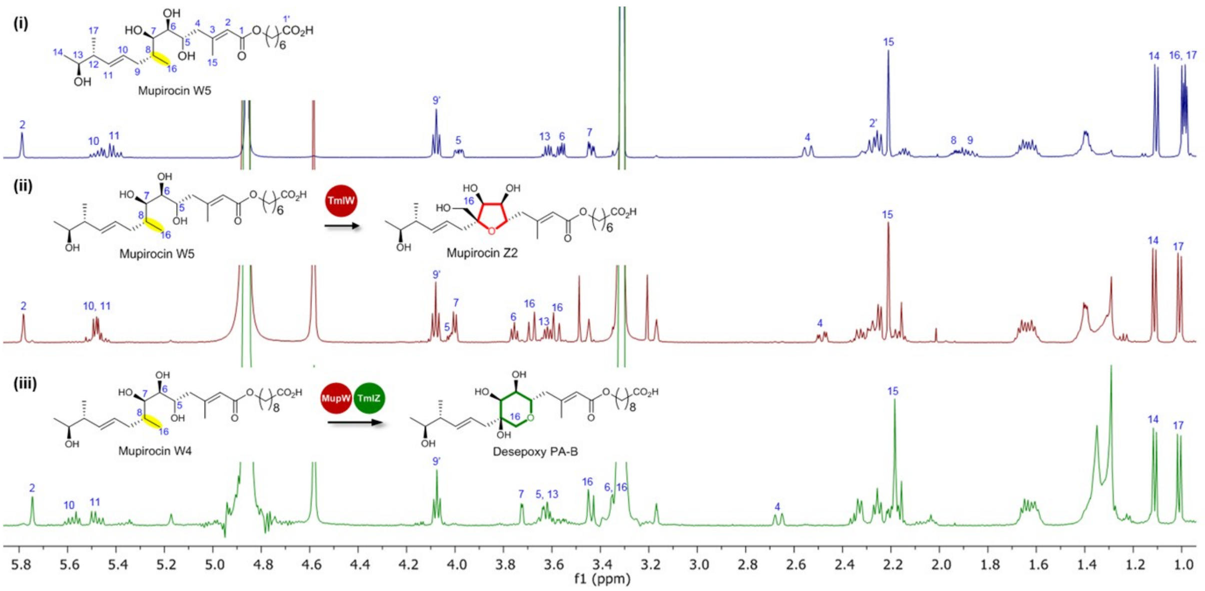

Figure S2. Whole-cell biotransformation with TmlW and TmlZ. (i) ^{1}H NMR spectrum of mupirocin W5 (CD_3OD, 700 MHz). (ii) ^{1}H NMR spectrum of product (CD_3OD, 700 MHz) from incubation of mupirocin W5 with *E. coli* BL21 overexpressing TmlW. (iii) ^{1}H NMR spectrum of product (CD_3OD, 700 MHz) from incubation of mupirocin W4 with *E. coli* BL21 overexpressing both MupW and TmlZ.

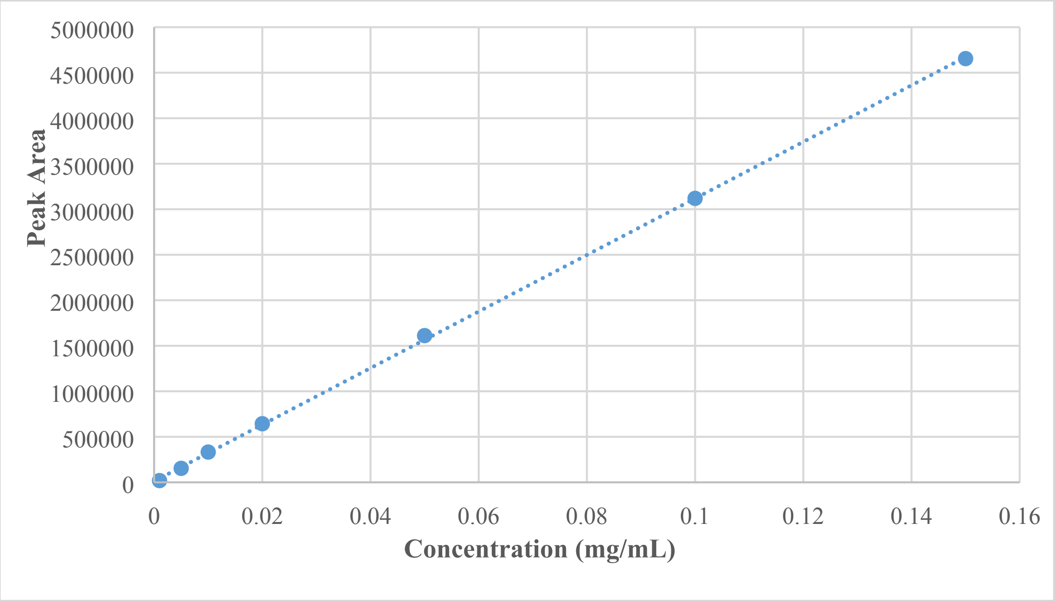

Figure S3. Standard curve of PA-C (**3**)

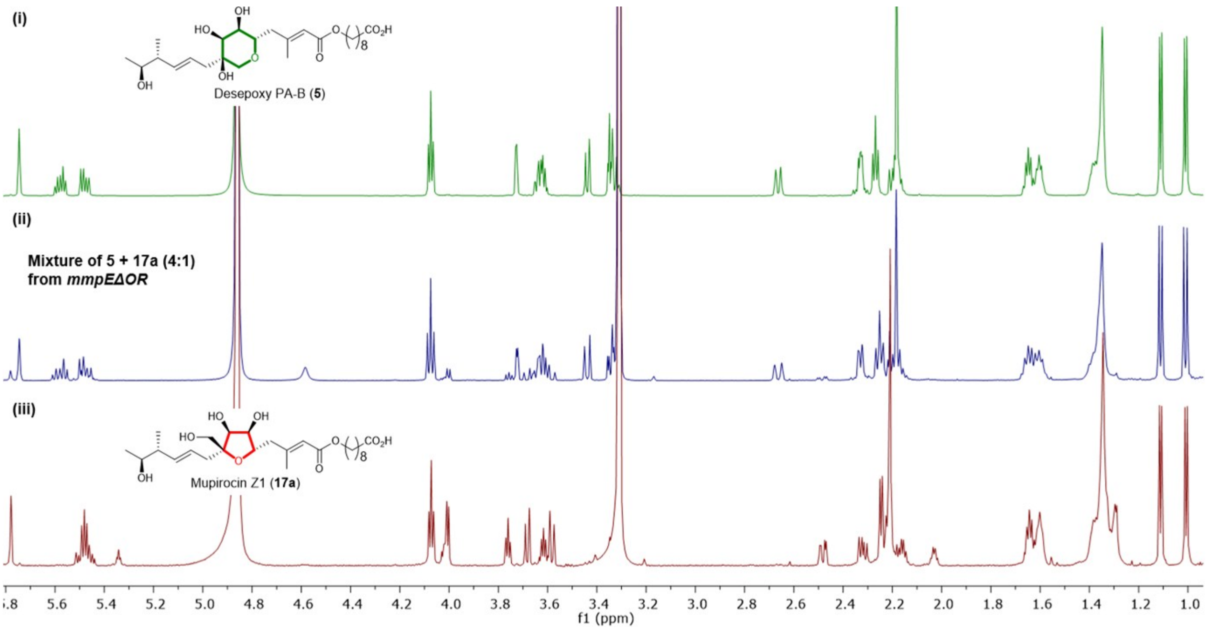

Figure S4. (i) ^{1}H NMR spectrum of desepoxy PA-B (**5**, CD_3OD, 500 MHz). (ii) ^{1}H NMR spectrum of mixture of **5** + **17a** (4:1, CD_3OD, 500 MHz) isolated from *mmpEΔOR* mutant of *P. fluorescens* NCIMB 10586. (iii) ^{1}H NMR spectrum of mupirocin Z1 (**17a**, CD_3OD, 700 MHz).

position	^{1}H NMR (500 MHz)	^{13}C NMR (125 MHz)
**1**		168.4 (C)
**2**	5.75 (1H, br s)	118.3 (CH)
**3**		159.1 (C)
**4**	2.66 (1H, br d, *J* = 14.4), 2.22 (1H, m)	44.1 (CH_2)
**5**	3.73 (1H, td, *J* = 9.3, 2.6 Hz)	76.1 (CH)
**6**	3.36 (1H, dd, *J* = 9.2, 3.2 Hz)	69.9 (CH)
**7**	3.84 (1H, dd, *J* = 3.3 Hz)	71.5 (CH)
**8**	1.77 (1H, m)	43.7 (CH)
**9**	2.18 (2H, m)	33.7 (CH_2)
**10**	5.44 (1H, m)	129.7 (CH)
**11**	5.44 (1H, m)	135.8 (CH)
**12**	2.16 (1H, m)	45.3 (CH)
**13**	3.61 (1H, qd, *J* = 6.3, 4.9 Hz)	72.1 (CH)
**14**	1.10 (3H, d, *J* = 6.3 Hz)	20.3 (CH_3)
**15**	2.19 (3H, s)	19.3 (CH_3)
**16**	3.78 (1H, dd, *J* = 11.5, 2.9 Hz)<br>3.51 (1H, dd, *J* = 11.5, 1.3 Hz)	65.7 (CH_2)
**17**	1.00 (3H, d, *J* = 6.9 Hz)	16.6 (CH_3)
**1'**		179.1 (C)
**2'**	2.28 (2H, br s)	36.5 (CH_2)
**3'**	2.28 (2H, br s)	29.6 (CH_2)
**4'**	5.48 (1H, m)	130.6 (CH)
**5'**	5.48 (1H, m)	131.7 (CH)
**6'**	2.03 (2H, m)	33.1 (CH_2)
**7'**	1.44 (2H, m)	27.0 (CH_2)
**8'**	1.64 (2H, m)	29.3 (CH_2)
**9'**	4.07 (2H, t, *J* = 6.6 Hz)	64.7 (CH_2)

Table S1. ^{1}H and ^{13}C NMR data of desepoxy PA-D (**19**) in CD_3OD

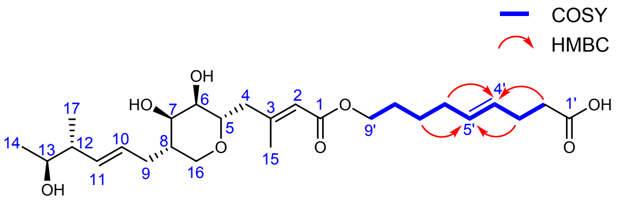

Figure S5. Key ^{1}H-^{1}H COSY and ^{1}H-^{13}C HMBC correlations of desepoxy PA-D (**19**)

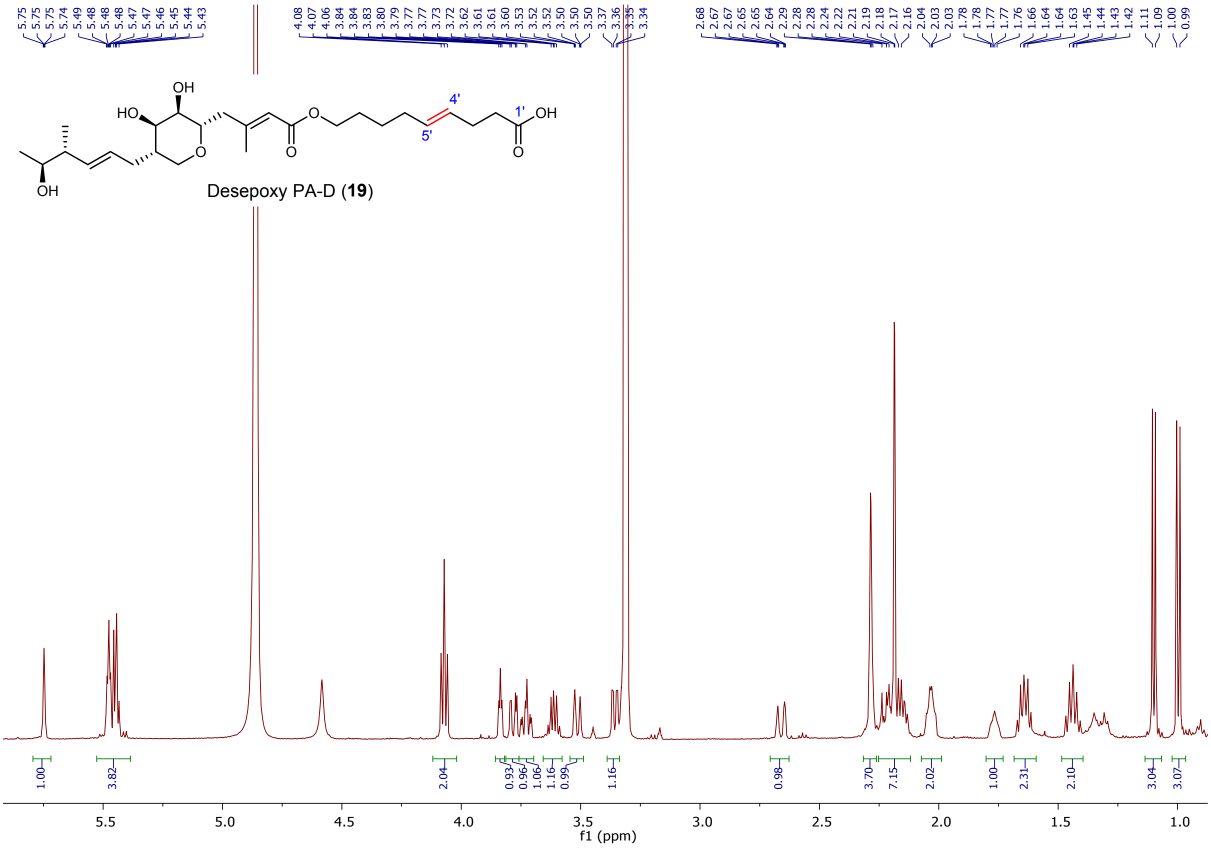

Figure S6. ^{1}H NMR spectrum of desepoxy PA-D (**19**, CD_3OD, 500 MHz)

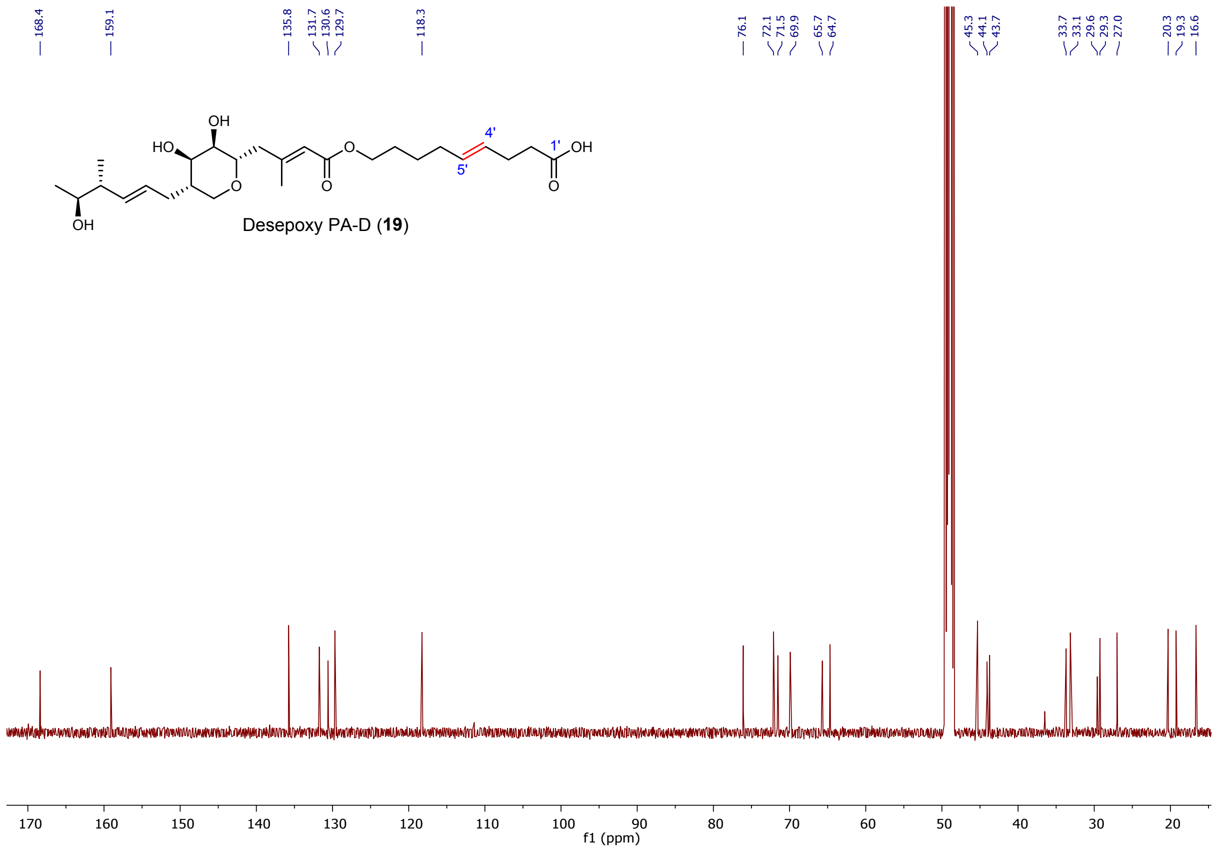

Figure S7. ^{13}C NMR spectrum of desepoxy PA-D (**19**, CD_3OD, 125 MHz)

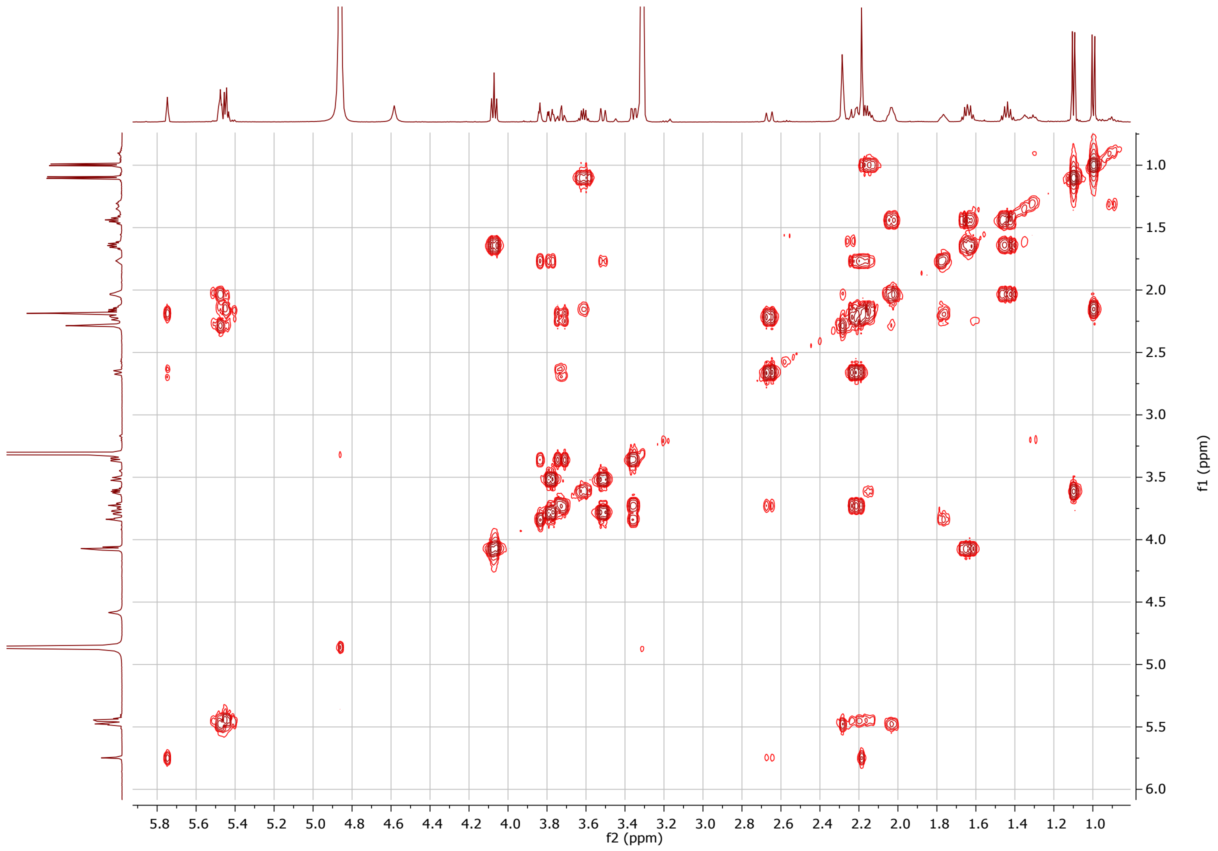

Figure S8. ^{1}H-^{1}H COSY spectrum of desepoxy PA-D (**19**)

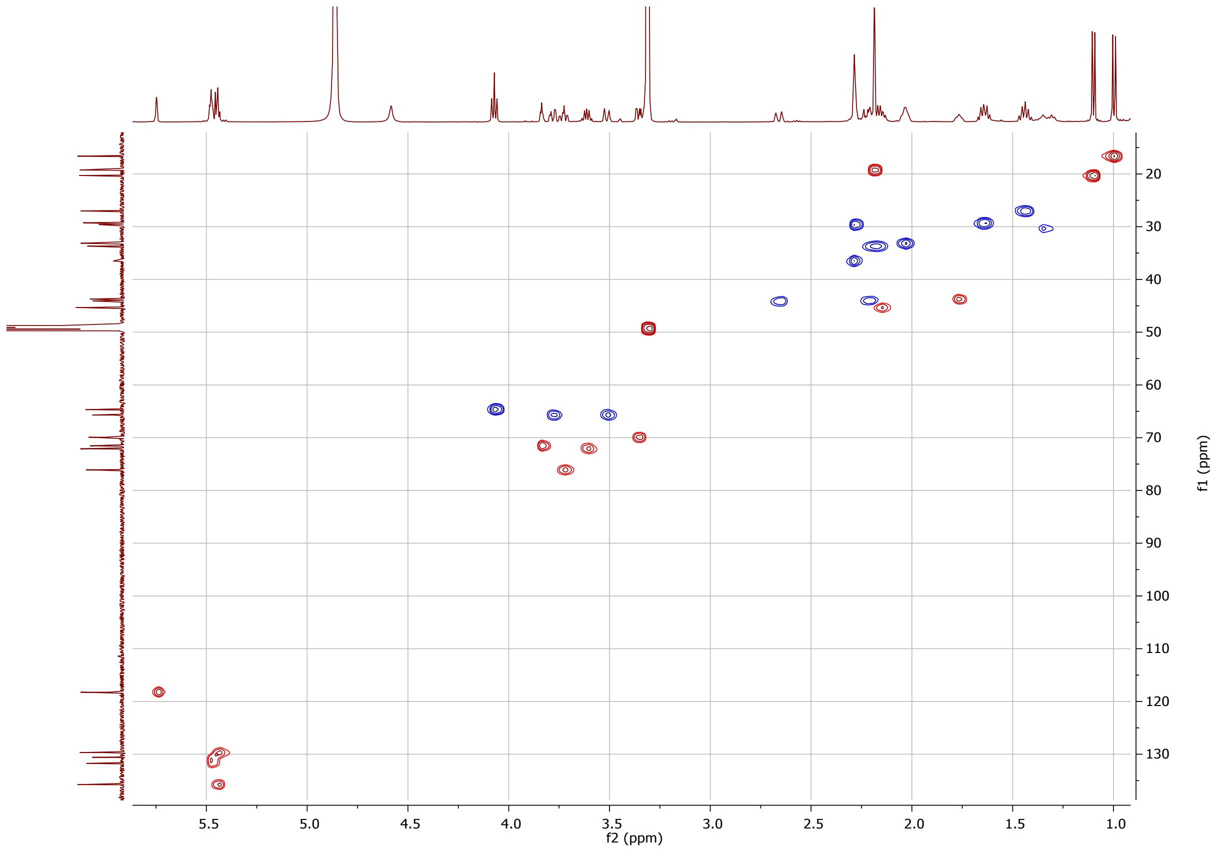

Figure S9. HSQC spectrum of desepoxy PA-D (**19**)

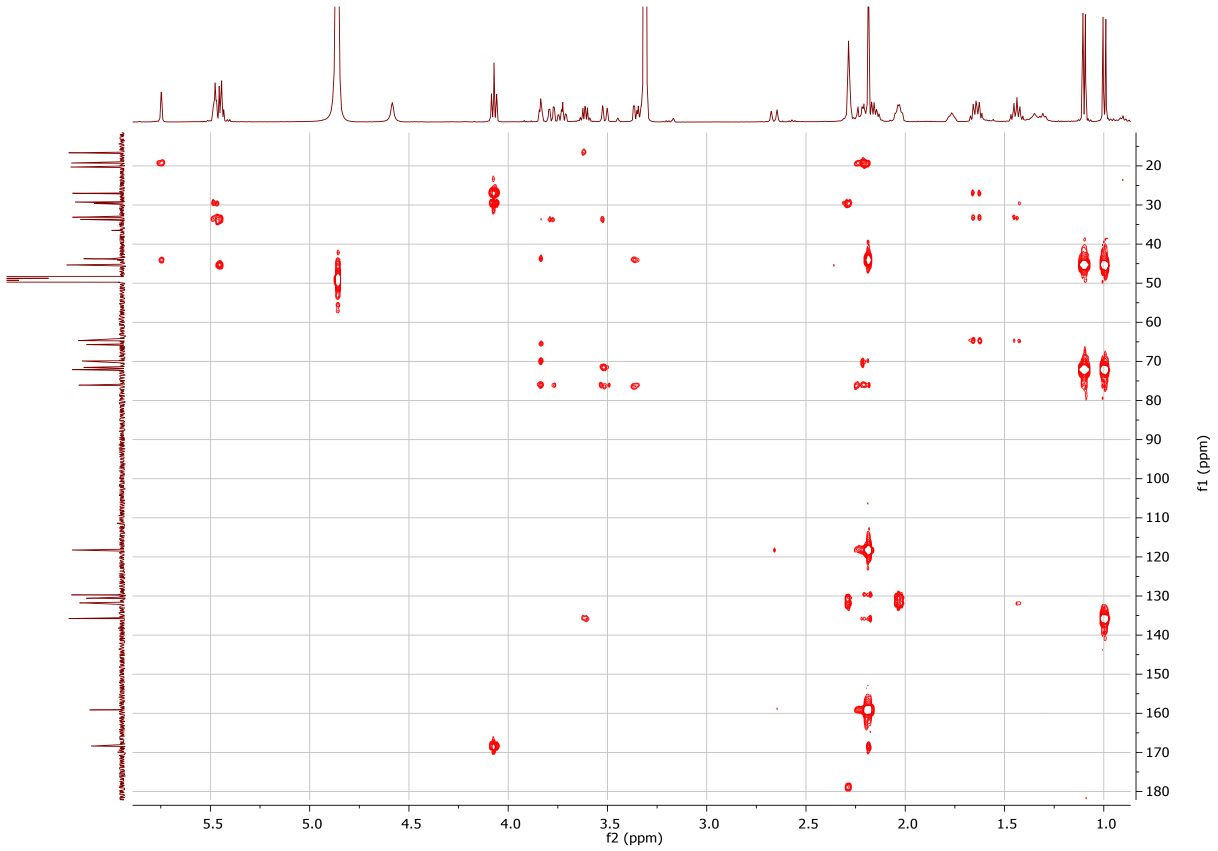

Figure S10. HMBC spectrum of desepoxy PA-D (**19**)
